# The Effect of Early and Delayed Harvest on Dynamics of Fermentation Profile, Chemical Composition, and Bacterial Community of King Grass Silage

**DOI:** 10.3389/fmicb.2022.864649

**Published:** 2022-04-07

**Authors:** Shihe Long, Xuefeng Li, Xianjun Yuan, Rina Su, Junxin Pan, Ye Chang, Mengli Shi, Zhihai Cui, Naixin Huang, Jian Wang

**Affiliations:** ^1^College of Animal Science and Technology, Hainan University, Haikou, China; ^2^College of Forestry, Hainan University, Haikou, China; ^3^Institute of Ensiling and Processing of Grass, Nanjing Agricultural University, Nanjing, China; ^4^State Key Laboratory of Grassland Agro-ecosystems, School of Life Sciences, Lanzhou University, Lanzhou, China

**Keywords:** king grass, silage, growth period, bacterial community, fermentation quality

## Abstract

The objective of this study was to assess the effect of harvesting time on the fermentation characteristics, chemical composition, and microbial community of king grass silage. King grass was harvested at three growth periods of 90 days (KN90S), 110 days (KN110S), and 130 days (KN130S); chopped into 2–3-cm particle size; and ensiled in polyethylene bags (20 × 30 cm). The fermentation quality and chemical composition of silages were analyzed after 1, 3, 7, 14, 30, and 60 days of ensiling. Bacterial community of silage ensiled for 60 days was profiled using next generation sequencing (NGS) technology. The KN110S showed the most extensive lactic acid (LA) fermentation during 7 days of fermentation compared to KN90S and KN130S. After 60 days of fermentation, the KN110S showed the lowest pH and the highest lactic acid content among the three treatments. The butyric acid and ammonia nitrogen contents of KN90S and KN130S were significantly greater than those of KN110S (*p* < 0.05). After a timespan of 60 days of ensiling, the bacterial community of king grass silage was predominantly populated by *Proteobacteria* in phylum level, whereas unclassified *Enterobacteriaceae* genus remained dominant in all silages. A higher relative abundance of *Clostridium* was observed in KN90S and KN130S, but not in KN110S, and greater abundance of *Lactococcus* appeared in KN110S and KN130S silages than KN90S. It is concluded that harvesting time had an important effect on the fermentation quality and microbial community of king grass silage.

## Introduction

King grass (*Pennisetum purpureum* Rich × *Pennisetum americanum*) is a major feed resource for ruminants in tropical regions due to its high nutritional values (Li et al., 2014) and can be harvested for five to eight times per year, with high biomass productions as much as 75–180 t of fresh matter (FM)/ha per year, which makes it an important potential resource in the development of animal feeding systems, production of biofuels, composts, or as a substrate for biodigestion (Botero-Londoño et al., 2021). In Hainan, utmost weather events such as typhoons and extreme rainfall frequently occurred in recent years, forcing farmers to adjust the forage harvest time to avoid agricultural losses. Therefore, proper preservation of king grass is one of the critical strategies to provide forage with consistent nutrients for ruminants and ensure a continuous supply of bioenergy production.

Ensiling is a prevailing forage preservation method for animal production due to its long storage duration, good palatability, and easy operation (McDonald et al., 1991). The provision of high-quality silage is the key to sustaining high productivity in intensive livestock pasture-based systems. The growth stage had been characterized as the main factor affecting silage quality because the dry matter (DM) production, ensilability, chemical composition, and nutritive value of forage are varied along with its change (Nazli et al., 2019). Early harvest crops were more suitable for ensiling because of higher water-soluble carbohydrates (WSC) (Stirling et al., 2021), but they showed a lower DM yield than delayed harvest (Xie et al., 2012). Besides, clostridial activity will be encouraged by the high moisture content of early harvested crops, resulting in considerable DM and energy losses (McDonald et al., 1991). Delayed harvest increases neutral detergent fiber (NDF) and acid detergent fiber (ADF) contents of crops, which is inconducive to preservation. At the same time, the WSC content tends to decrease and slow down the fermentation, retarding the decrease of pH necessary for efficient preservation (Kalač, 2011).

Complex microbial interactions directly determine the natural preservation process in terms of ensiling. To get an insight into the microbial community of silages is critical to distinguish valuable microorganisms and understand the underlying scientific basis for optimal silage making (Nishino, 2015; Guo et al., 2018). In this context, multiple molecular techniques have been applied to investigate the microbial ecology of silages (McAllister et al., 2018), among them is the next generation sequencing (NGS) technique, an advanced tool used for identifying microbial community in many crops and forages (Zhao et al., 2017; Keshri et al., 2019; Xu et al., 2019; Bai et al., 2021). However, it is still unclear whether different growth stages affect bacterial community in king grass silage.

It is hypothesized that the growth stage affected the chemical composition and microbial community of king grass, which in turn affected its fermentation quality. In the present study, we estimate the response of chemical composition and microbial community of king grass silage at different growth stages related to harvest time, which could provide a theoretical basis for improving king grass silage *via* control of harvest time.

## Materials and Methods

### Raw Materials and Silage Preparation

The king grass (cultivar Reyan No. 4) was planted in the experimental field of Hainan University (Haikou, China, 20°06′N, 110°32′E) on June 20, 2020. After 90, 110, and 130 days of growth, three plots of the forage were harvested at noon to avoid the variation in the diurnal rhythm of nutrients induced by photosynthesis and respiration of herbage. Fresh forage was immediately transported to the laboratory, cut into a theoretic length of 20–30 mm using a forage chopper, and mixed evenly. Approximately 250 g of fresh mix was ensiled in polyethylene bags (20 × 30 cm; Dongguan Bojia Packing Products Co., Ltd., China) and vacuumed tightly using a vacuum sealer (YS-10 L; Fujian Jiamei Machinery Co., Ltd., China). Each treatment uses 18 silage bags (six ensiling durations × three replicates). All bags were stored at an ambient temperature and sampled after 1, 3, 7, 14, 30, and 60 days of ensiling. Bacterial community was profiled in silage ensiled for 60 days. Additionally, a subsample of fresh materials was sampled for the analysis of chemical characteristics and microbial counts.

### Fermentation Parameter, Chemical Composition, and Microbial Enumeration

Silage samples of 20 g were homogenized with 180 ml of distilled water and stored at 4°C overnight, followed by filtering through four layers of cheesecloth. The filtrate was used to determine pH and the concentrations of organic acid and ammonia nitrogen (AN). The pH was measured immediately using a glass electrode pH meter (PHS-3C; INESA Scientific Instrument, Shanghai). The organic acids, including lactic acid (LA), acetic acid (AA), propionic acid (PA), and butyric acid (BA), were measured using high-performance liquid chromatography (LC20A, Shimadzu, Ltd., Tokyo, Japan), equipped with a UV detector (SPD-M10AVP) (column, Carbomix H-NP; mobile phase, 2.5 mmol/l of H_2_SO_4_, pH 2.6; flow rate, 0.6 ml/min; temperature, 55°C; injection volume, 5 μl; and SPD, 210 nm). The AN concentration was analyzed as described by Weatherburn (1967). To determine the DM content, a subsample of fresh and silage was oven-dried at 65°C for 72 h. The dried samples were then ground through a 1-mm screen by a feed grinder (YBF-2500; Wande, China) for the following chemical composition analyses. Crude protein (CP) concentration was quantified by an automatic Kjeldahl nitrogen analyzer (K9860, Shandong Haineng Scientific Instrument Co., Ltd., China). The concentrations of NDF and ADF were measured as reported by Van Soest et al. (1991), and WSC were measured according to Owens et al. (1999).

Using the pour plate technique for microbial enumeration (Man et al., 1960), 20 g of fresh samples were blended with 180 ml of sterilized saline (8.5 g/l) and serially diluted to 10–fold. Lactic acid bacteria (LAB) were anaerobically incubated at 37°C for 48 h on de Man, Rogosa and Sharpe agar (Solarbio Life Sciences Co., Ltd., Beijing, China). Coliform bacteria were aerobically incubated at 37°C for 48 h on Violet Red Bile agar (Solarbio Life Sciences Co., Ltd., Beijing, China). Yeasts and molds were aerobically cultured at 25°C for 72 h on Rose Bengal agar (Solarbio Life Sciences Ltd. Co., Beijing, China).

### Microbial Community Analysis

After 60 days of fermentation, 20 g of silage sample was homogenized with 100 ml of sterile sodium chloride solution at 120 for 15 min, followed by filtering through four layers of cheesecloth. Total DNA was extracted by TIANamp Bacteria DNA isolation kit (DP302–02, Tiangen Biotech Co., Ltd., Beijing, China) according to the manufacturer’s protocols. The quality of the extracted DNA was checked by a NanoPhotometer N50 UV-Vis spectrophotometry (Implen GmbH, Germany). All extracted DNA samples were frozen at −80°C for further analysis. The V3–V4 region of 16S rRNA was amplified with primers 341F (5′-CCTACGGGNGGCWGCAG-3′) and 805R (5′-GACTACHVGGGTATCTAATCC-3′). The reads were merged into a sequence using PEAR (version 0.9.8), removing the bases with a mass value below 20 at the tail of reads through PRINSEQ (version 0.20.4) to obtain clean data. Operational taxonomic unit (OTU) clustering was performed with 97% of similarity through the Usearch program (version 11.0.667).^1^ The Ribosome Database Project (RDP) algorithm was used to classify the representative sequences of each OTU at different levels. The alpha diversity including Shannon index, Chao richness estimator, Ace, and Good’s coverage index was calculated using Mothur (version 1.43.0).^2^ Venn diagrams (version 1.6.20) was used to construct the Venn diagram, and the principal coordinates analysis (PCoA) presented in this study was conducted with the VEGEN package in R software (version 3.6.0) (Dixon, 2003).

### Statistical Analysis

Microbial populations were estimated as colony-forming units (CFU)/gram of forage, and logarithmic conversion was performed before statistical analysis. The fermentation profiles of the silages were analyzed in a completely randomized block design with 3 × 6 factorial treatment arrangements with three replicates per treatment. The prime effect of the growth period, ensiling duration, and their interactions were analyzed by the general linear model (GLM) procedure of SAS 9.3 software (SAS Institute Inc., 2019). Significance was declared at *p* < 0.05 and tendencies at 0.05 < *p* ≤ 0.10.

## Results

### Chemical and Microbial Composition of King Grass Before Ensiling

The chemical and microbial composition of king grass before ensiling are depicted in Table 1. The growth period had a significant effect (*p* < 0.01) on contents of DM, WSC, CP, ADF, and NDF, as well as LAB, molds, yeasts, and coliform bacteria numbers. The WSC content of king grass ranged from 73.8 to 94.7 g/kg of DM. The undesirable microorganism (e.g., mold, yeast, and coliform bacteria) counts of KN110S were the lowest among three groups. The readings of LAB (3.8–4.4 log_10_ CFU/g of FM), ADF (385.8–439.5 g/kg of DM), and NDF (647.3–710.7 g/kg of DM) increased (*p* < 0.05), whereas CP (92.3–66.7 g/kg of DM) content decreased (*p* < 0.05) as the growth time prolonged.

**TABLE 1:** Chemical characteristics and attached microorganisms of king grass before ensiling.

Item^1^	Silages^2^			SEM^3^	*p*-value
	KN90S	KN110S	KN130S		
DM (g/kg of FM)	170.7^b^	174.2^b^	205.9^a^	0.61	<0.01
WSC (g/kg of DM)	80.6^b^	73.8^b^	94.7^a^	3.37	<0.01
CP (g/kg of DM)	92.3^a^	85.0^a^	66.7^b^	4.06	<0.01
NDF (g/kg of DM)	647.3^a^	684.3^b^	710.7^c^	9.56	<0.01
ADF (g/kg of DM)	385.8^b^	425.0^a^	439.5^a^	8.39	<0.01
LAB (log_10_ CFU/g of FM)	3.8^c^	3.9^b^	4.4^a^	0.09	<0.01
Mold (log_10_ CFU/g of FM)	3.1^a^	2.5^b^	3.1^a^	0.11	<0.01
Yeast (log_10_ CFU/g of FM)	3.6^b^	3.3^b^	4.3^a^	0.17	<0.01
Coliform bacteria (log_10_ CFU/g of FM)	4.2^a^	3.1^b^	4.2^a^	0.19	<0.01

*Means with different superscript letters in the same row (a–c) differ significantly from each other (p < 0.05). ^1^DM, dry matter; FM, fresh matter; WSC, water-soluble carbohydrates; CP, crude protein; ADF, acid detergent fiber; NDF, neutral detergent fiber. ^2^King grass silage harvested at different growth periods. KN90S, king grass silage harvested at growth periods of 90 days; KN110S, king grass silage harvested at growth periods of 110 days; KN130S, king grass silage harvested at growth periods of 130 days. ^3^SEM, standard error of the mean.*

### Fermentation Characteristics and Chemical Composition of King Grass Silage

As shown in Tables 2, 3 the ensiling duration and growth period had a significant effect (*p* < 0.01) on all fermentative parameters except for LA/AA, and there were interactions on the levels of pH, DM, WSC, CP, ADF, LA, AA, LA/AA, PA, BA, and AN. The DM contents of all king grass silage gradually decreased with ensiling time, and the marked decline (*p* < 0.05) was observed in KN90S. The CP concentrations of KN90S, KN110S, and KN130S (91.7 vs. 73.1 g/kg of DM; 86.4 vs. 80.2 g/kg of DM; 66.0 vs. 52.5 g/kg of DM, respectively) statistically reduced (*p* < 0.05) after 60 days of ensiling, whereas the ADF (average of 396.0 vs. 440.7 g/kg of DM) and NDF (average of 690.8 vs. 718.4 g/kg of DM) contents increased. For KN110S, the CP concentration decreased slightly to a smaller extent than those in KN90S and KN130S. The WSC concentrations in KN90S (71.4 vs. 28.6 g/kg of DM), KN110S (65.2 vs. 43.9 g/kg of DM), and KN130S (79.3 vs. 58.8 g/kg of DM) were dramatically reduced within the early stage of fermentation.

**TABLE 2:** Changes in the chemical composition of king grass silage during ensiling.

Item^1^	Silages^2^	Ensiling time (days)						SEM^3^	*p*-value		
				
		1	3	7	14	30	60		*E*	*G*	*E* × G
DM (g/kg of FM)	KN90S	161.6^ab^	152.8^abC^	138.4^b^*^cC^*	118.2*^d^*^b^	128.1^c^*^dB^*	126.4^c^*^dC^*	3.08	<0.01	<0.01	<0.01
	KN110S	174.3^ab^	168.6^a^*^bB^*	167.6^a^*^bB^*	168.7^a^*^bA^*	162.4^ca^	159.5^cb^				
	KN130S	191.3^aa^	187.8^a^*^bA^*	198.8^aa^	177.3^b^*^cA^*	170^ca^	177.6^b^*^cA^*				
WSC (g/kg of DM)	KN90S	71.4^a^	28.6^bc^	24.2^b^	13.0^c^	10.2^ca^	5.6^c^	3.33	<0.01	<0.01	<0.01
	KN110S	65.2^a^	43.9^bb^	31.0^c^	16.0*^d^*	6.3*^e^*^b^	6.0*^e^*				
	KN130S	79.3^a^	58.8^ba^	36.0^c^	14.5*^d^*	8.7*^deAB^*	7.6*^e^*				
CP (g/kg of DM)	KN90S	91.7^aa^	85.3^ba^	84.6^ba^	81.1^ba^	71.8^cb^	73.1^cb^	1.55	<0.01	<0.01	<0.01
	KN110S	86.4^aa^	84.8^a^*^bA^*	81.4^a^*^bB^*	79.5^ba^	79.6^ba^	80.2^ba^				
	KN130S	66.0^ab^	67.5^ab^	64.3^ac^	63.9^ab^	53.9^bc^	52.5^bc^				
ADF (g/kg of DM)	KN90S	376.2^cb^	385.3^cc^	416.6^bb^	435.7^a^	436.2^a^	441.5^a^	3.25	<0.01	<0.01	<0.01
	KN110S	400.1^ba^	413.5^bb^	407.9^bb^	439.2^a^	433.7^a^	431.4^a^				
	KN130S	411.7^ba^	437.4^aa^	429^a^*^bA^*	451.3^a^	446.4^a^	449.3^a^				
NDF (g/kg of DM)	KN90S	674.2^cb^	714^aa^	718.9^aa^	737.2^aa^	726.2^aa^	721.3^aa^	3.12	<0.01	<0.01	0.02
	KN110S	684.1^b^	686.1^b^	685.9^b^	698.9^b^	686.5^b^	699.1^b^				
	KN130S	714.2^ba^	708.6^ba^	733.5^aa^	720.7^a^*^bAB^*	738.1^aa^	734.7^aa^				

*Means with different superscript letters in the same row (a–e) or column (A–C) differ significantly from each other (p < 0.05). ^1^DM, dry matter; FM, fresh matter; WSC, water-soluble carbohydrates; CP, crude protein; ADF, acid detergent fiber; NDF, neutral detergent fiber. ^2^King grass silage harvested at different growth periods. KN90S, king grass silage harvested at growth periods of 90 days; KN110S, king grass silage harvested at growth periods of 110 days; KN130S, king grass silage harvested at growth periods of 130 days. ^3^SEM, standard error of the mean.*

**TABLE 3:** Changes in the fermentation quality of king grass silage during ensiling.

Item^1^	Silages^2^	Ensiling time (days)						SEM^3^	*p*-value		
		1	3	7	14	30	60		*E*	*G*	*E* × *G*
pH	KN90S	5.6^ab^	5.7^abA^	5.9^aa^	5.3^bcA^	5.2^bcA^	5.0^cAB^	0.07	<0.01	<0.01	<0.01
	KN110S	5.8^a^	5.0^bb^	4.5^cc^	4.5^cc^	4.5^cb^	4.6^cb^				
	KN130S	6.0^a^	5.8^ba^	5.7^bb^	5.1^cb^	5.3^ca^	5.2^ca^				
LA (g/kg of DM)	KN90S	2.2^cb^	1.5^cb^	2.1^cc^	10.0^bb^	11.2^bb^	11.5^bb^	1.10	<0.01	<0.01	<0.01
	KN110S	6.4^ca^	14.5^ba^	21.4^aa^	23.2^aa^	26.0^aa^	22.0^aa^				
	KN130S	1.9^ab^	4.4^cb^	8.1^bb^	12.6^ab^	9.1^bb^	9.8^bb^				
AA (g/kg of DM)	KN90S	0.6^cc^	10.0^bb^	9.7^b^	15.5^a^	19.5^a^	14.6^a^	0.66	<0.01	<0.01	<0.01
	KN110S	14.3^a^	15.7^a^	12.7	12.7	15.8	16.9				
	KN130S	6.3^bb^	9.3^abB^	8.6^ab^	10.7^ab^	11.8^a^	12.7^a^				
LA/AA	KN90S	3.8^aa^	0.2^bc^	0.2^bb^	0.6^bb^	0.6^bb^	0.9^bb^	0.13	0.01	0.06	<0.01
	KN110S	0.4^ab^	0.9^abA^	1.7^aa^	1.9^aa^	1.8^aa^	1.3^abA^				
	KN130S	0.3^ab^	0.5^bcB^	1.1^abA^	1.2^a^*^AB^*	0.8^abcB^	0.8^abcB^				
PA (g/kg of DM)	KN90S	0.6^ca^	0.8^bcA^	1.1^abA^	1.4^a^	0.9^bcB^	1.1^ab^	0.06	<0.01	<0.01	<0.01
	KN110S	0.2^bb^	0.2^bb^	0.4^bb^	0.9^a^	0.9^ab^	0.8^a^				
	KN130S	0.2^cb^	0.4^cb^	0.4^cb^	0.9^b^	1.4^aa^	1.3^a^				
BA (g/kg of DM)	KN90S	2.6*^e^*	4.4^c^*^d^*	3.6^c^*^d^*	5.1^bc^	7.0^bAB^	10.2^aa^	0.35	<0.01	<0.01	<0.01
	KN110S	2.8^b^	2.6^b^	3.8^a^	4.2^a^	4.6^ab^	4.7^ab^				
	KN130S	2.3^c^	4.4^bc^	6.1^abC^	7.9^ab^	8.4^abA^	9.9^aa^				
AN (g/kg of TN)	KN90S	19.0*^d^*	61.1^ca^	95.8^b^*^cA^*	129.0^ba^	221.6^aa^	244.7^aa^	10.12	<0.01	<0.01	<0.01
	KN110S	13.5*^d^*	34.6^cdB^	51.8^bcB^	68.4^abB^	86.1^ac^	89.4^ab^				
	KN130S	27.4*^e^*	53.2*^d^*^eAB^	79.2^cdA^	102.4^cAB^	189.2^bb^	232.9^aa^				

*Means with different superscript letters in the same row (a–e) or column (A–C) differ significantly from each other (p < 0.05). ^1^LA, lactic acid; AA, acetic acid; PA, propionic acid; BA, butyric acid; AN, ammonia nitrogen; TN, total nitrogen; LA/AA, the ratio of lactic acid to acetic acid. ^2^King grass silage harvested at different growth periods. KN90S, king grass silage harvested at growth periods of 90 days; KN110S, king grass silage harvested at growth periods of 110 days; KN130S, king grass silage harvested at growth periods of 130 days. ^3^SEM, standard error of the mean.*

Different extents of LA fermentation occurred among the three groups. The pH value of KN110S reached 4.5 after 7 days of ensiling, while the pH value of KN90S and KN130S remained above 5. AA contents of KN110S and KN130S presented an increasing trend during ensiling, and LA/AA values decreased from days 14 to 60, while a significant decrease in LA/AA exhibited in KN90S from days 1 to 3. A significant increase (*p* < 0.05) of PA, BA, and AN content was observed in king grass silage on day 60 compared with day 1, and the final PA contents of the three treatments were around 1.1 g/kg of DM. KN110S appeared to have a slower accumulation of BA and AN concentrations than KN90S and KN130S during ensiling and the lowest final BA (4.7 vs. 10.2 and 9.9 g/kg of DM) and AN (89.4 vs. 244.7 and 232.9 g/kg of TN) at 60 days.

### Bacterial Diversity of King Grass Silage After 60 Days of Ensiling

**FIGURE 1:**
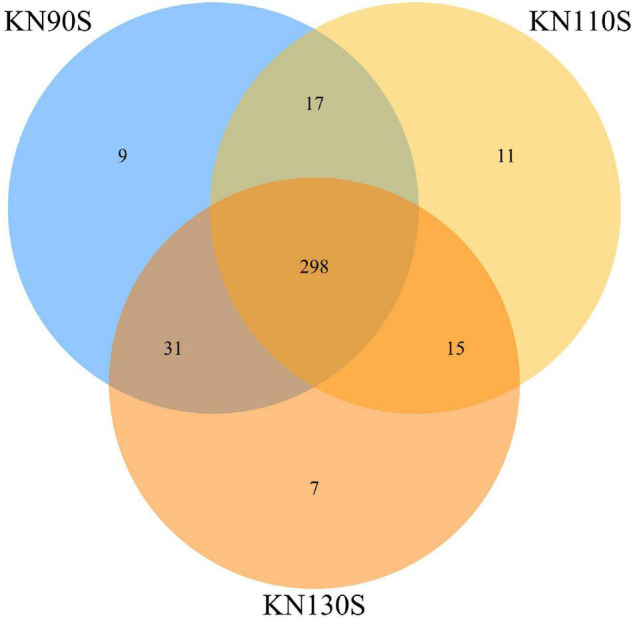
Venn diagram of the core OTUs of king grass silage (KN90S, king grass silage harvested at growth periods of 90 days; KN110S, king grass silage harvested at growth periods of 110 days; KN130S, king grass silage harvested at growth periods of 130 days; OTU, operational taxonomic unit).

**FIGURE 2:**
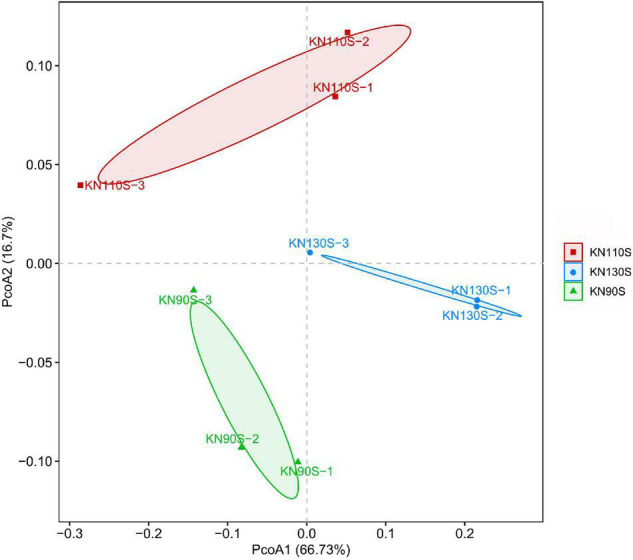
Principal coordinates analysis (PCoA) of the bacterial community for king grass silages (KN90S, king grass silage harvested at growth periods of 90 days; KN110S, king grass silage harvested at growth periods of 110 days; KN130S, king grass silage harvested at growth periods of 130 days).

The bacterial alpha diversity of king grass silage is shown in Table 4. A total of 500,278 sequences of bacteria were obtained using NGS technology. The Good’s coverage values of all groups were 0.99, and a non-significant difference was observed in OTUs, Shannon, Chao1, Ace, and Simpson indexes among KN90S, KN110S, and KN130S. The core OTUs were displayed in the Venn diagram (Figure 1). The overlapping OTUs for the bacterial communities among king grass silages at three different harvest times were 298, accounting for 91.69% of total OTUs. The bacterial community of king grass silages can be clearly demonstrated by PCoA. As shown in Figure 2, principal component 1 (PCoA1) and component 2 (PCoA2) can be used to explain 66.7 and 16.7% of the total variance, respectively, indicating that the bacterial composition of silages from different growth periods could be well separated from each other.

**TABLE 4:** Alpha diversity of bacterial diversity for king grass silage.

Silages^1^	Number of reads	Observed OTUs^2^	Shannon	Chao	Ace	Simpson	Good’s coverage
KN90S	37,653.33^ab^	292	2.02	341.11	349.36	0.30	0.99
KN110S	37,933.00^a^	273	1.99	330.95	329.48	0.34	0.99
KN130S	32,404.33^b^	271	1.94	329.91	340.51	0.38	0.99
SEM^3^	882.410	5.541	0.063	9.882	8.242	0.030	0.001
*p*-value	0.07	0.30	0.84	0.88	0.64	0.53	0.83

*Means with different superscript letters in the same column (a–b) differ significantly from each other (p < 0.05). ^1^KN90S, king grass silage harvested at growth periods of 90 days; KN110S, king grass silage harvested at growth periods of 110 days; KN130S, king grass silage harvested at growth periods of 130 days. ^2^OTUs, observed taxonomic units. ^3^SEM, standard error of the mean.*

**FIGURE 3:**
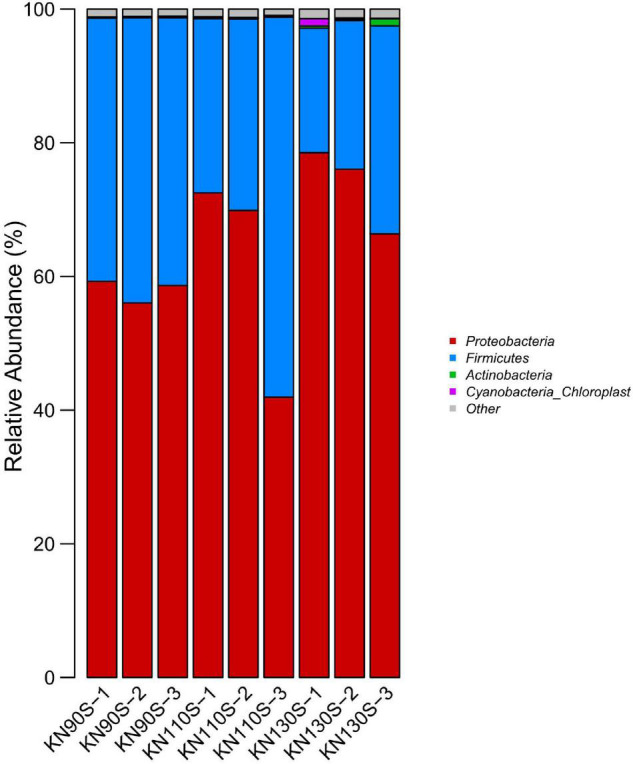
Differences in the bacterial community of king grass silage at the phylum level (KN90S, king grass silage harvested at growth periods of 90 days; KN110S, king grass silage harvested at growth periods of 110 days; KN130S, king grass silage harvested at growth periods of 130 days).

**FIGURE 4:**
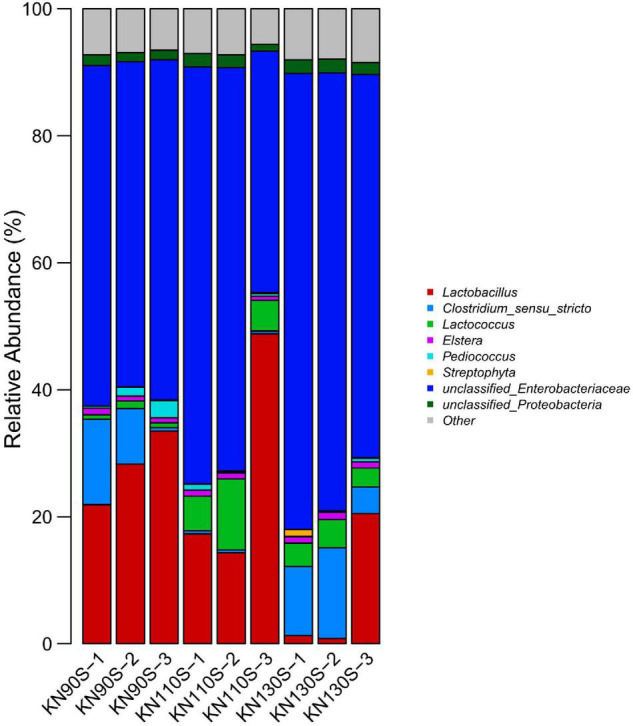
Differences in the bacterial community of king grass silage at the genus level (KN90S, king grass silage harvested at growth periods of 90 days; KN110S, king grass silage harvested at growth periods of 110 days; KN130S, king grass silage harvested at growth periods of 130 days).

The bacterial communities at the phylum level revealed that *Proteobacteria* was the most abundant phylum (average of 65.17%) in king grass silage (Figure 3). Conversely, *Firmicutes* only occupied an average of 34.83% of the population. The bacterial composition of king grass silage at the genus level was also analyzed to better understand the bacterial community of king grass silage (Figure 4). The top three genera of the KN110S silage were unclassified-*Enterobacteriaceae* (55.0%), *Lactobacillus* (27.9%), and *Lactococcus* (6.9%), while unclassified-*Enterobacteriaceae* (52.8 and 67.0%), *Lactobacillus* (28.0 and 7.6%), and *Clostridium* (7.4 and 9.8%) were the first three dominating genera observed only in KN90S and KN130S. A greater abundance of *Lactobacillus* than *Lactococcus* and *Pediococcus* (average of 22.794.15, and 0.80%, respectively) appeared in king grass silage. The relative abundance of *Lactococcus* in KN90S, KN110S, and KN130S was 0.9, 6.9, and 3.7%, respectively. A higher relative abundance of *Clostridium* was found in KN90S (7.4%) and KN130S (9.8%) silages, which was consistent with the greater BA and AN content of these groups.

## Discussion

### Chemical and Microbial Composition of King Grass

Sufficient epiphytic LAB (10^5^ CFU/g FM) and WSC contents (5% DM) of fresh material were considered as essential factors for assuring the acceptable fermentation quality (McDonald et al., 1991). The WSC content of king grass in the present study may provide enough fermentable substrate for the extensive fermentation. However, the number of LAB was lower than the theoretical requirement for good preservation. Previous findings reported that the number of microorganisms on the surface of plants varied with the growth periods (Xie et al., 2012; Lounglawan et al., 2014), and the epiphytic LAB increased with delayed harvest dates (Müller, 2009). The chemical composition and nutritional value of forages change along with the growth period (Nazli et al., 2019). The decline in CP and the increase in ADF and NDF contents in king grass with the extension of growth days were in line with that of Botero-Londoño et al. (2021) in king grass with different cutting ages. They found a linear increase in fiber levels with the age of cutting and a significant decrease in crude protein levels. The decrease in CP was due to an increase in the proportion of stems (Ribeiro et al., 2014), while the increase in fiber concentration was due to the lignification process as harvest dates progressed (Darby and Lauer, 2002).

### Fermentation Characteristics and Chemical Composition of King Grass Silage

The loss of dry matter during ensiling will reduce the nutritive value of silage. DM showed significant decreases after 60 days of ensiling, which is in agreement with the report by Wang et al. (2020) in Italian ryegrass silages. Savoie et al. (2002) reported that mature corn silage (108 days of growth) produced less effluent (1.41 vs. 2.47% of the original wet mass) than premature corn silage (94 days of growth) on a laboratory scale. The decrease in DM content during ensiling was due to the activity of undesirable bacteria, which metabolized soluble carbohydrates to CO_2_, causing DM losses (Ávila et al., 2014), while the severe decline in DM in KN90S during 7 days of fermentation was due to effluent production. High-moisture silage often bears a high risk of effluent production, leading to high DM loss (Zhao et al., 2021), and the peak effluent of silage typically occurs within 10 days after ensiling given the time required for plant cell to rupture (Gebrehanna et al., 2014). This is consistent with the view of Savoie et al. (2002), who found that the amount of daily effluent collected from the timothy silage was relatively high within 7 days of ensiling, and the effluent flow declined in subsequent days.

The degradation of protein is inevitable when silage is made, as a result a combination of plant enzymes and microbial metabolism (McDonald et al., 1991). According to the higher BA and AN content, statistically reduced CP concentrations in this study reflect that king grass underwent extensive fermentation of *Clostridium butyricum* and protein degradation during ensiling (Kung et al., 2018). This might be explained by the fact that silage with a high moisture content bears a great risk of extensive clostridial fermentation, resulting in a large amount of protein hydrolysis and BA occurrence (He et al., 2020a). A relatively low degree of CP degradation in KN110S could be explained by a rapid drop in pH value. A previous study reported that protein breakdown and high concentrations of AN in silages are typically caused by a slow drop in pH (Ogunade et al., 2018). Similarly, Li et al. (2020) found that fast acidification of silage was conducive to preventing clostridial fermentation. Likewise, the protein degradation in alfalfa silage has been effectively inhibited by inoculated *L. plantarum* on account of the fast drop in pH that inhibits the activity of proteolytic bacteria (Filya et al., 2007). Fiber can hardly be degraded in silage due to its complex fibrous structures (Nair et al., 2020). In present study, we found an increased trend of ADF and NDF during ensiling, which was similar to those reported by Wang C. et al. (2019), but it was contrary to the study of Zi et al. (2021) who found a numerically declined ADF and NDF contents of king grass silage without additives during fermentation. This may be due to the loss of DM during ensiling, increasing the relative levels of ADF and NDF (Ferrero et al., 2019). Furthermore, fermentation of sugars into CO_2_ by microbial metabolism at the ensiling process can lead to increases in the fibrous fraction of silage (Ávila et al., 2008), and the metabolism of fermented substrates to metabolites (mainly organic acids) by microorganisms anaerobically can also increase the contents of ADF and NDF. The consumption rate of WSC correlates with the extent of fermentation. The rapid decline of WSC during the initial stage of ensiling indicates that all king grass underwent extensive fermentation.

Rapid acidification of silage depends on LA production by LAB, which is imperative to prevent the propagation of early undesirable microorganisms and the loss of nutrients (Liu et al., 2016). The most rapid decrease in pH and increase in LA content were found in KN110S, followed by KN130S and KN90S. This might be attributed to the lowest attached undesirable microorganisms in KN110S, and the lower BA concentration of KN110S also confirms the extensive LA fermentation of KN110S silages (Shao et al., 2007). However, the final pH value of all silages was still above the ideal silage pH of 4.2 at day 60. The finding was consistent with a previous work by Li D. et al. (2019) in several tropical forage silage after 60 days of fermentation including king grass. The rates of diffusion of WSC from intact and ruptured cells into the aqueous phase are rather important than the total amounts in the crop for silage making (McDonald et al., 1991), considering that there was adequate fermentable substrate of fresh king grass in this study. Distinct from temperate crops, the higher incorporation of air in the ensiling and the restriction of cell breakdown and juice release due to the properties of a tropical grass with coarse porosity and stemmy structures delayed the onset of the fermentation phase during the very early stage of the ensiling by extending respiration and aerobic microbial activity (Shao et al., 2005), causing the higher loss of WSC, which is detrimental to subsequent anaerobic lactic acid fermentation to drop pH (Dunière et al., 2013). A previous study has reported that AA-type fermentation was considered the primary pattern in tropical silage (Nishino et al., 2012), and AA was the main fermentation product in various tropical silages including paspalum, stylo, and cassava (Li D. et al., 2019; Li M. et al., 2019). The rapid increase in AA concentration and the marked decrease in LA/AA in KN90S silage are also reported by Yuan et al. (2020) in Napier grass silage from 3 to 5 days of ensiling. The production of AA may be partly related to the metabolism of AA-producing bacteria (e.g., *Enterobacter*) and the ability of homofermentative LAB to convert LA anoxically into AA and 1,2-propanediol (Oude Elferink et al., 2001). The production of PA in silages was likely caused by several microorganisms such as *Propionibacterium* sp. and *Clostridium propionicum* (Santos et al., 2016). A significant increase in the PA content was observed in king grass silage on day 60 compared to day 1, although this may not be of practical significance because of their low concentration. The final PA content of the three treatments were around 1.1 g/kg of DM, which is consistent with previous reports that observed a close PA content in king grass silage without additives (Li D. et al., 2019; Zi et al., 2021).

### Bacterial Diversity of King Grass Silage

This study revealed the relative abundance and diversity of bacteria of king grass silage from different growth stages. The analysis of the alpha diversity of the bacterial community revealed the variance of the microbial community. The coverage values of all samples were 0.99, suggesting that the sequencing had adequately reflected the profile of the bacterial community. The highly overlapping OTUs in the present study reflect that many similar microbes were involved in silage. PCoA clearly demonstrates the variance of the microbial community by the clear separation of the three treatments, which indicates that growth periods had a remarkable effect on the bacterial community of king grass silage and that ensiling could be the main factor affecting fermentation. The most dominant phyla in the 60-day king grass silage were *Proteobacteria*, followed by *Firmicutes*. *Proteobacteria* in silage is comprised of the families *Enterobacteriaceae*, *Sphingomonadaceae*, *Xanthomonadaceae*, and *Aurantimonadaceae* and the genera *Sphingomonas*, *Pantoea*, *Pseudomonas*, and *Stenotrophomonas* (Ogunade et al., 2018). This phenomenon was inconsistent with previous studies reporting that the dominant phylum was shifted from *Proteobacteria* to *Firmicutes* along with the ensiling process (Xu et al., 2017; Yuan et al., 2020), although Wang et al. (2020) reported a dominant *Proteobacteria* (62.45%) phylum at 60 days in ensiling sorghum forages. To our knowledge, the ensiling conditions favored the selection of species belonging to *Firmicutes* because these species thrive under low pH and anaerobic conditions (Dong et al., 2019). This may be related to the high pH environment (>4.60), which is not low enough to enhance the shift from *Proteobacteria* to *Firmicutes* phylum after fermentation. *Enterobacteria* is a non-spore forming, facultative anaerobe that can ferment LA to AA and other products (Santos et al., 2016) and was usually outcompeted by *Lactobacillus* species with a decrease in pH during ensiling (Parvin et al., 2010), but we found a high relative abundance of the unclassified-*Enterobacteriaceae* that appeared in 60-day silage, which could be attributed to the high moisture of typical tropical grass. Wang Y. et al. (2019) suggested that a lower abundance of *Enterobacter* spp. might be connected to the high DM content of *Moringa oleifera* leaf (448 g/kg of FM) silages. Li D. et al. (2019) reported that *Enterobacter* was the predominant genus in high-moisture tropical forage including king grass, paspalum, and stylo silages, accompanied by high AA production. He et al. (2020a) made a similar report in which *Enterobacter* (39.3%) was the dominant genus of corn straw silage with high moisture content, and its relative abundance decreased from 39.3 to 28.9% and the AA content decreased significantly when the moisture content was lowered by mixing corn stalk with *Bauhinia variegata* flower. The high relative abundance of unclassified-*Enterobacteriaceae* in all silage samples might account for the high AA content (Nishino et al., 2012; Wang et al., 2020). Previous reports suggested that cocci such as *Lactococcus*, *Leuconostoc*, *Pediococcus*, and *Enterococcus* initiated lactic acid fermentation at the early stage of fermentation (Cai et al., 1998), and they were outcompeted by more acid-tolerant *Lactobacillus* species with the decline of pH as the ensiling time prolonged (Graf et al., 2016), which might partially explain the higher level of *Lactobacillus* than *Lactococcus* and *Pediococcus* in this study. The portion of *Lactococcus* remains high after ensiling, especially in the KN110S and KN130S silages. Xu et al. (2017) determined the bacterial community in corn stover silage by high-throughput sequencing and found that the abundance of *Lactococcus* in natural silage was higher than that of inoculated silage (5.69% vs. 0.37%), and the pH value of the inoculated group decreased more efficiently in the early stages of ensiling. Mu et al. (2020) detected greater relative abundances of *Lactococcus* in untreated mixed silage of amaranth and rice straw compared to those treated with additives. Likewise, Wang Y. et al. (2019) reported a minor presence of *Lactococcus* (less than 1%) in *Moringa oleifera* leaf silage with a pH below 4.2 after 60 days of ensiling. As aforementioned, a relatively slowly drop in pH and higher 60-day silage pH might explain the greater relative abundance of *Lactococcus* in KN110S and KN130S silages, whereas a low relative abundance of *Lactococcus* in KN90S may be due to the loss of soluble sugars caused by the effluent, resulting in a deficit of substrates that obstructed competitive colonization of *Lactococcus* in the early stage of fermentation. *Clostridium* was often detected in silages, and its presence is undesirable since it can utilize carbohydrates and proteins as a substrate for their growth and ferment LA to BA, resulting in DM and energy losses (McDonald et al., 1991). The higher relative abundance of *Clostridium* presented in this study is consistent with the greater BA and AN content. This might be because high-moisture grasses always face a greater risk of *Clostridium* fermentation (He et al., 2020b; Mu et al., 2020). *Clostridium* inhibition in KN110S (0.5%) supported the speculation that clostridial fermentation was restricted by a faster rate of pH decline compared to KN90S and KN130S.

## Conclusion

The KN110S silage displayed a relatively higher LA content, lactic acid bacteria abundance at the genus level, lower pH values, contents of AN and BA, and abundance of *Clostridium* compared with the KN90S and KN130S silage after ensiling. The results of this study indicated that harvesting date plays a paramount role in the fermentation parameter and microbial community of king grass silage. We recommend that king grass with a growth period of 110 days provides silage with better fermentation quality.

## Data Availability Statement

The datasets presented in this study can be found in online repositories. The names of the repository/repositories and accession number(s) can be found below: https://www.ncbi.nlm.nih.gov/, PRJNA800375.

## Author Contributions

SL and JW designed the experiments. SL, JP, YC, MS, ZC, and NH conducted the experiments. SL, YC, and MS analyzed the data. SL wrote the manuscript. SL, XY, XL, and RS reviewed the manuscript. All authors contributed to the article and approved the submitted version.

## Conflict of Interest

The authors declare that the research was conducted in the absence of any commercial or financial relationships that could be construed as a potential conflict of interest.

## Publisher’s Note

All claims expressed in this article are solely those of the authors and do not necessarily represent those of their affiliated organizations, or those of the publisher, the editors and the reviewers. Any product that may be evaluated in this article, or claim that may be made by its manufacturer, is not guaranteed or endorsed by the publisher.

## Footnotes

1
http://drive5.com/uparse/

2
http://www.mothur.org/wiki/Classify.seqs
